# Minimum number of synaptic vesicles for the initiation of a single action potential at *C. elegans* neuromuscular junction

**DOI:** 10.17912/micropub.biology.000316

**Published:** 2020-10-06

**Authors:** Lili Chen, Ya Wang, Shangbang Gao

**Affiliations:** 1 Key Laboratory of Molecular Biophysics of the Ministry of Education, College of Life Science and Technology, Huazhong University of Science and Technology, Wuhan 430074, China

**Figure 1:**
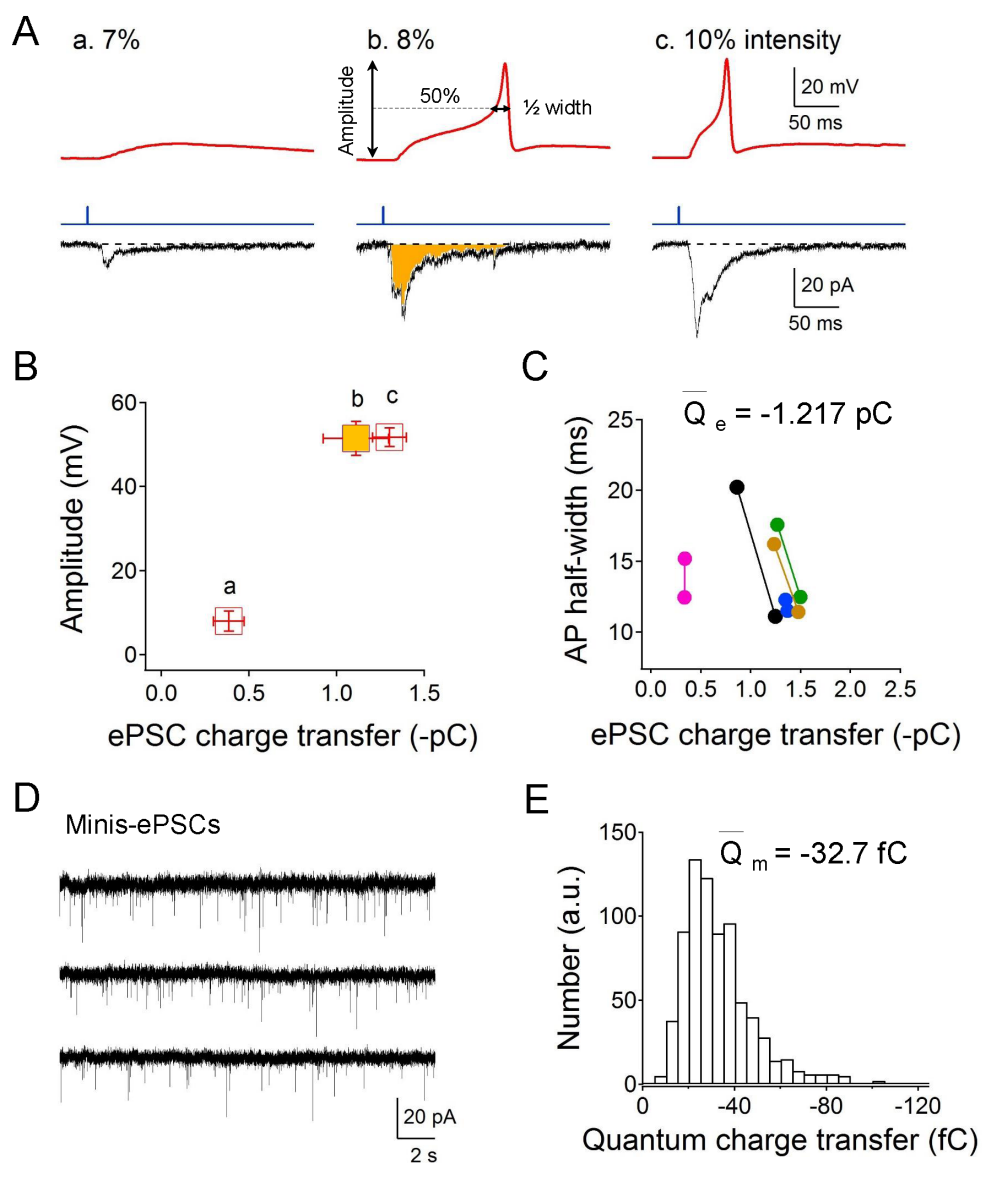
**(A)** Representative membrane potential response and excitatory postsynaptic currents (EPSCs) under gradually increased blue light irradiation (1 ms, 7%, 8%, and 10% of full intensity 8 mW/mm^2^). Data were collected with at least 45 seconds interval under current-clamp (*upper*, holding at 0 pA) and voltage-clamp (*bottom*, holding at -30 mV), respectively. The yellow area denotes the transferred charge. **(B)** Quantification of the amplitude of evoked membrane potentials with correlated total EPSCs charge. a, 7% light intensity; b, 8% light intensity; c, 10% light intensity (n=6). **(C)** Correlation of the AP half-width with the total charge from the same preparations under paired light stimulation (*upper left*, 8% light intensity; *bottom right*, 10% light intensity). The average charge evoked by 8% light intensity (~0.64 mW/mm^2^) for initiating a single action potential is -1.217 ± 0.115 pC (n=6). **(D)** Representative miniature-EPSCs traces holding at -30 mV. **(E)** The minis charge distribution and average charge transfer of a single event is -32.7 ± 2.7 fC (n=6).

## Description

The action potential (AP) is the basic signaling unit in various crucial physiological processing, for instance, in neurotransmission, muscle contraction, and glandular secretion (Koch, 1990). The classic model animal, *Caenorhabditis elegans (or C. elegans)*, with a simple and compact nervous system, conservatively employs the calcium-mediated all-or-none APs for odor response in AWA olfactory neurons (Liu **et al.*,* 2018), as well as for muscle contraction in body wall muscles (Gao and Zhen, 2011; Liu **et al.*,* 2011) and pharyngeal muscles (Davis **et al.*,* 1999). Plateau potentials were also observed in ASE and RMD neurons (Goodman **et al.*,* 1998; Mellem **et al.*,* 2008; Lockery **et al.*,* 2009; Lockery and Goodman, 2009), though the underlying roles in specific behavior are still elusive. Either in neurons or in muscles, the action potential firing is dependent on the excitatory pre-synaptic vesicles release. The minimum number of the presynaptic vesicles to elicit a single action potential in *C. elegans* has not been reported before. Here, by the combination of optogenetics with *in-vivo* patch clamping technology, we demonstrated that at least approximately 37 excitatory acetylcholinergic vesicles are required for the initiation of an action potential at post-synaptic body wall muscles.

We used the transgenic strain ZX460 with *zxIs6* that expresses the optogenetic protein, ChR2 (H134R), in all excitatory cholinergic motor neurons. By gradually increasing the irradiated blue light intensity (from 1-10% of the full intensity 8 mW/mm^2^), graded electrical responses and eventually all-or-none APs were evoked at the neuromuscular junction. Graded increased excitatory postsynaptic currents (EPSCs) were also recorded. The used holding potential of -30 mV is the reversal potential of postsynaptic ionotropic GABA receptor and therefore the GABAergic inhibitory PSCs could not be recorded (Gao and Zhen, 2011; Maro **et al.*,* 2015). Specifically, as the light intensity reached 8% of the full intensity (~0.64 mW/mm^2^), solid action potentials could be evoked (Fig 1A). Consistent with the all-or-none principle, the amplitude of the evoked muscular action potentials was no longer changed even with stronger light stimulation (Fig 1B, bc). The AP half-width, however, was slightly shortened and was correlated with higher EPSCs charge transfer (Fig 1C). The average smallest EPSCs quantity of electric charge is -1.217 pC for the elicitation of an action potential in muscle cells. To evaluate the single vesicle charge transfer, we measured miniature-EPSCs holding at -30 mV (Fig 1D). The average miniature-EPSCs quantity of electric charge is -32.7 fC (Fig 1E), thus the calculated minimum number of cholinergic vesicles is 37 by dividing the quanta charge into the smallest EPSCs charge.

## Methods

**Strains and Culturing Conditions**

Strain ZX460: *zxIs6 [Punc-17::ChR2(H134R)::YFP + lin-15(+)]* was cultured in the dark at 22℃ on OP50-seeded Nematode Growth Medium (NGM) plates (Brenner, 1974). Plates containing all trans-retinal were prepared by spreading 300 μl of OP50 culture mixed with 0.25 μl of 100 mM all trans-retinal stock (dissolved in ethanol) (Liewald **et al.*,* 2008).

**Electrophysiology**

The dissection of *C. elegans* was described previously (Richmond **et al.*,* 1999, Gao and Zhen, 2011). Briefly, one-two days old hermaphrodite adults were glued to a sylgard-coated cover glass covered with bath solution. Animals were immobilized on Sylgard^®^ 184 Silicone Elastomer (Dow Corning)-coated glass coverslips using tissue adhesive glue (Histoacryl^® ^Blue, Braun). The curved dorsal side of the animal was dissected using sharpened tungsten or glass needle. After clearing the viscera by suction through a glass pipette, the cuticle flap was turned and gently glued down using WORMGLU (GluStitch Inc.) to expose the neuromuscular system. The muscle cells were patched using fire-polished 4−6 MΩ resistant borosilicate pipettes (World Precision Instruments, USA). Membrane potential and currents were recorded in the whole-cell configuration by a Digi-Data 1440A and a MultiClamp 700A amplifier using the Clampex 10 software, and data were processed with Clampfit 10.2 (Molecular Devices). Data were digitized at 10 kHz and filtered at 2.6 kHz. Light stimulation of *zxIs6* was performed with an LED lamp (KSL-70; RAPP OptoElectronic) at a wavelength of 470 nm (full intensity 8 mW/mm^2^), controlled by the Axon amplifier software.

In this study, each cell recording is from one animal unless otherwise noted. ‘n’ means the recorded animal number. Usually, three membrane potentials, and three EPSCs data evoked by different light intensities (7%, 8%, 10%) from a muscle cell were collected and analyzed.

The recording solutions used in this study: the pipette solution contains (in mM) K-gluconate 115; KCl 25; CaCl_2_ 0.1; MgCl_2_ 5; BAPTA 1; HEPES 10; Na_2_ATP 5; Na_2_GTP 0.5; cAMP 0.5; cGMP 0.5, pH7.2 with KOH, ~320 mOsm. The bath solution consists of (in mM) NaCl 150; KCl 5; CaCl_2_ 5; MgCl_2_ 1; glucose 10; sucrose 5; HEPES 15, pH7.3 with NaOH, ~330 mOsm. Leak currents were not subtracted. All chemicals were from Sigma. Experiments were performed at room temperatures (20−22℃).

**Statistical Analysis**

Data analysis and graphing were performed using Excel 2013 (Microsoft), Igor Pro 6.21 (Wavemetrics), and Clampfit 10.2 (Molecular Devices). All data are presented as mean ± SEM.

## Reagents

ZX460 *zxIs6 [Punc-17::ChR2(H134R)::YFP; lin-15+] V*.
